# A quality improvement project to assess the use of preventative measures against acute alveolar osteitis

**DOI:** 10.1038/s41405-019-0019-7

**Published:** 2019-06-26

**Authors:** Toby Andrew Mummery, Miriam John, Susan Mary Stokes

**Affiliations:** 1 School of Clinical Dentistry, University of Sheffield, Sheffield, UK; 2 LINCymru Clinical Lead, Quality Improvement Skills Training Section, Health Education and Improvement Wales, Cardiff, UK; 3 Dental Educator Quality Improvement, Dental Postgraduate Section Health Education Improvement Wales, Cardiff, UK

**Keywords:** Dental patient assessment, Dental patient management, Oral surgery

## Abstract

**Aims:**

A quality improvement project was conducted in a General Dental Practice environment. The aim was to reduce the rate of Acute Alveolar Osteitis, which was locally found to be at 19.4%.

**Methods:**

A range of quality improvement tools were utilised to determine and measure potential interventions, and the results from the initial Plan-Do-Study-Act cycle utilising perioperative 0.2% Chlorhexidine as a preventative method are presented.

**Results:**

The use of perioperative 0.2% Chlorhexidine mouthwash showed an absolute risk reduction of 6.2%.

**Discussion:**

Generalisation from the results is highly dependent on local factors, although the favourable reduction in acute alveolar osteitis and cost savings found supported the project.

**Conclusions:**

This project highlights the strengths of Quality Improvement methodologies in implementing and assessing changes to improve service provision and patient outcomes.

## Background

The Dental Teaching Unit, Port Talbot Resource Centre (DTU) is located in South Wales, and provides in-hours access (IHA) sessions for NHS Direct, as well as providing routine care and treatment for the local area. Both patient groups present with an overall high dental treatment need and varying levels of dental neglect. Routine treatment plans frequently include planned extractions, and IHA patients often require emergency extractions to manage pain and infection.

Acute Alveolar Osteitis (AAO) is a relatively common post-extraction complication, which sees a failure of healing characterised by the loss of the clot from the socket, superficial infection and acute pain and discomfort that is often described as worse than the previous toothache.^1^

Anecdotally and observationally, a high number of DTU patients develop AAO. To evidence this, a 1-week review was undertaken which showed that a total of 26 extractions were conducted, with a total of five (19.2%) re-attending within 2 weeks due to AAO. A later large-scale audit of 1129 extraction appointments found 219 (19.4%) reattended due to AAO. As some IHA patients will utilise NHS Direct again, rather than contacting the practice for follow-up care, the rate of AAO is feasibly higher than this observation indicates.

The rate of AAO reported within the literature ranges between 1 and 4% and up to 30% for third molar removal^2^ with a number of risk factors have been identified, including complexity of extraction, smoking status, and use of the contraceptive pill.^3^ Current local practice is that patients are consented for extraction. This includes the risk of AAO with reference to identified risk factors. Additional preventative interventions are not currently utilised with cases of AAO being management by irrigation with saline and Alvogyl (an antiseptic and analgesic dressing) following occurrence.

Methods for preventing AAO focus on two areas—modifiable patient behaviours such as smoking, with referral to smoking cessation services routinely offered in clinic and post-operative instructions recommending smoking abstinence for 48 h post extraction, and protective interventions including the utilisation of systemic or topical antibacterial and antibiotics agents such as amoxicillin and Chlorhexidine (CHx).^3^ The efficacy of these interventions is varied, and the overall evidence base is limited, however, it is recognised that the ability to modify patient behaviours is restricted, and the use of antibacterial agents, whilst efficacious in some studies in reducing AAO in third molar extractions has a weaker evidence base for routine extractions.^4^ Furthermore, with increasing focus on antibiotic resistance and appropriate prescribing, coupled with the risks of anaphylaxis, the routine prescribing of antibiotics for preventative purposes is increasingly contraindicated or not supported by current guidelines.^3^

The “Model for Improvement” was selected as the most appropriate quality improvement framework for this project.^5^ This facilitated a progressive environment with changes being central to the overall aim. This stands in contrast to clinical audit (CA), where recent evidence has undermined the validity of using CA as the impetus of improvement with one study estimating only 5% of audits led to any change in practice.^6,7^ This was supported by the utilisation of the Sustainability Model, which represents a useful tool in planning an intervention as it highlights 10 key factors which influence sustaining change.^8^ These cover the process, staff and organisational factors, and are weighted to highlight the more central role that some of these factors play.^9^

The project was undertaken as part of the Silver Improving Quality Together curriculum, supported by the Quality Improvement Skills Training (QIST) Section of Health Education and Improvement Wales.

## Aims and objectives

Based on the higher-than-expected rate of AAO, the stated aim of this project was to reduce the rate of AAO in adult patients within the DTU undergoing extraction by 10% within a 3-month period.

Objectives were: to determine the rate of AAO within the practice, to identify potential associated factors, to assess the efficacy of an identified intervention in reducing the local rate of AAO, and to determine the long-term sustainability of the intervention within the clinical environment.

## Methods

**Fig. 1:**
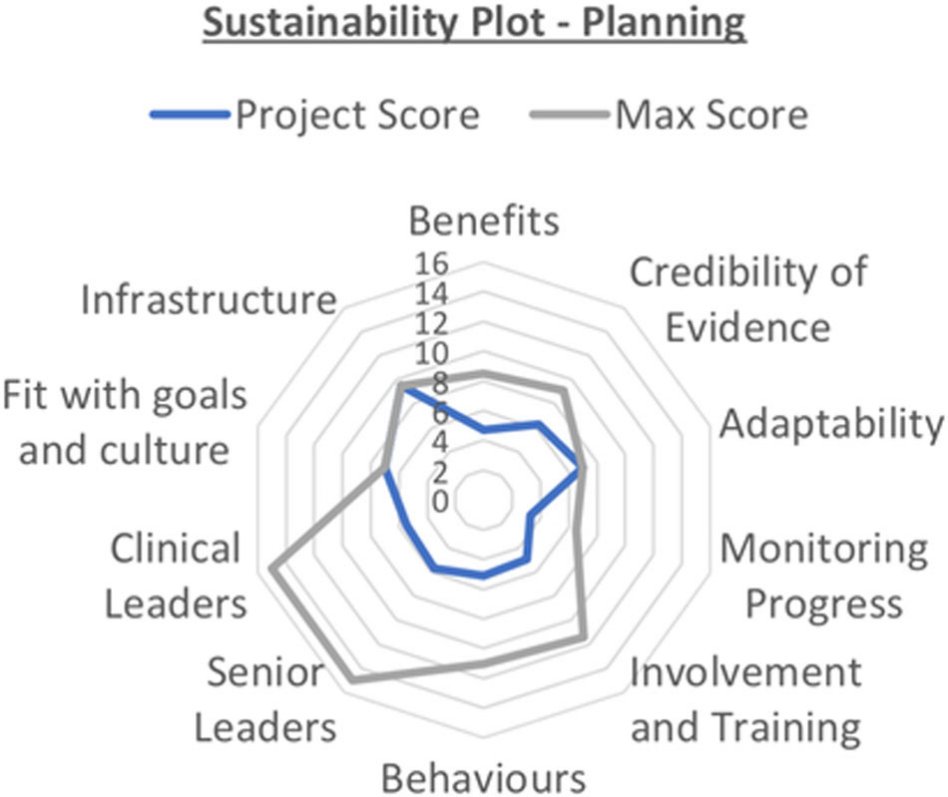
Sustainability model-planning phase

Contextual elements of relevance were broadly considered under the sustainability of the project. Within the scope of this project, a Sustainability Model was developed in the planning phase to highlight potential factors to change moving forward (see Fig. 1). This highlighted the importance of considering and engaging stakeholders, particularly clinical and senior leaders, throughout the project.

**Fig. 2:**
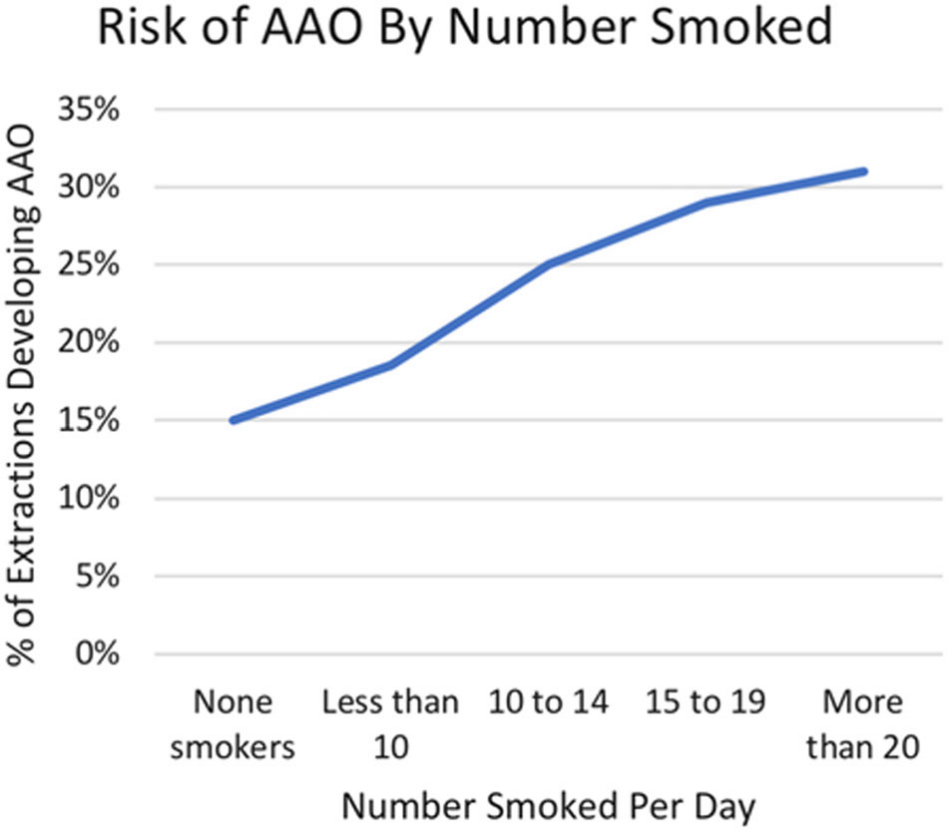
Local risk of AAO by quantity smoked per day

Under the Model for Improvement change is more central than in CA and falls under the umbrella of the Plan-Do-Study-Act approach (PDSA). In assessing the literature and local situation, it was unclear whether a root cause could be identified for the increased local rate; as such, extraction notes were reviewed to collect information regarding recognised risk factors (1129 extraction appointments, over a 1-year period). This showed that the overall risk within the unit at 19.4%, with recognised risks such as wisdom tooth removal and difficult extractions (where flaps had been raised or bone removed) increasing the risk. Of specific interest was a positive correlation with smoking (see Fig. 2), with smoking more than 20/day increasing the risk of AAO to over 30%. This was considered highly relevant to the higher-than-expected local level given that 49% of patients included in the review were active smokers. This is significantly higher than the reported local rate of smoking within the Port Talbot area at 25%, which is already twice the Welsh national average.^10^ That being said, smoking cessation advice was already routine and smoking abstinence following extraction was already recommended. Furthermore, as a significant proportion of the patients attended through IHA, the scope to provide continued cessation support was limited and smoking was considered an unmodifiable risk factor for AAO in this case.

Based on these results, a number of interventions were considered to reduce the rate of AAO and a simple ease/benefit analysis was performed to consider which of these were most feasible for an initial intervention.^11^ As the use of antibiotic agents was deemed inappropriate, the use of an oral antiseptic (CHx) was identified as the focus of the intervention.

**Fig. 3:**
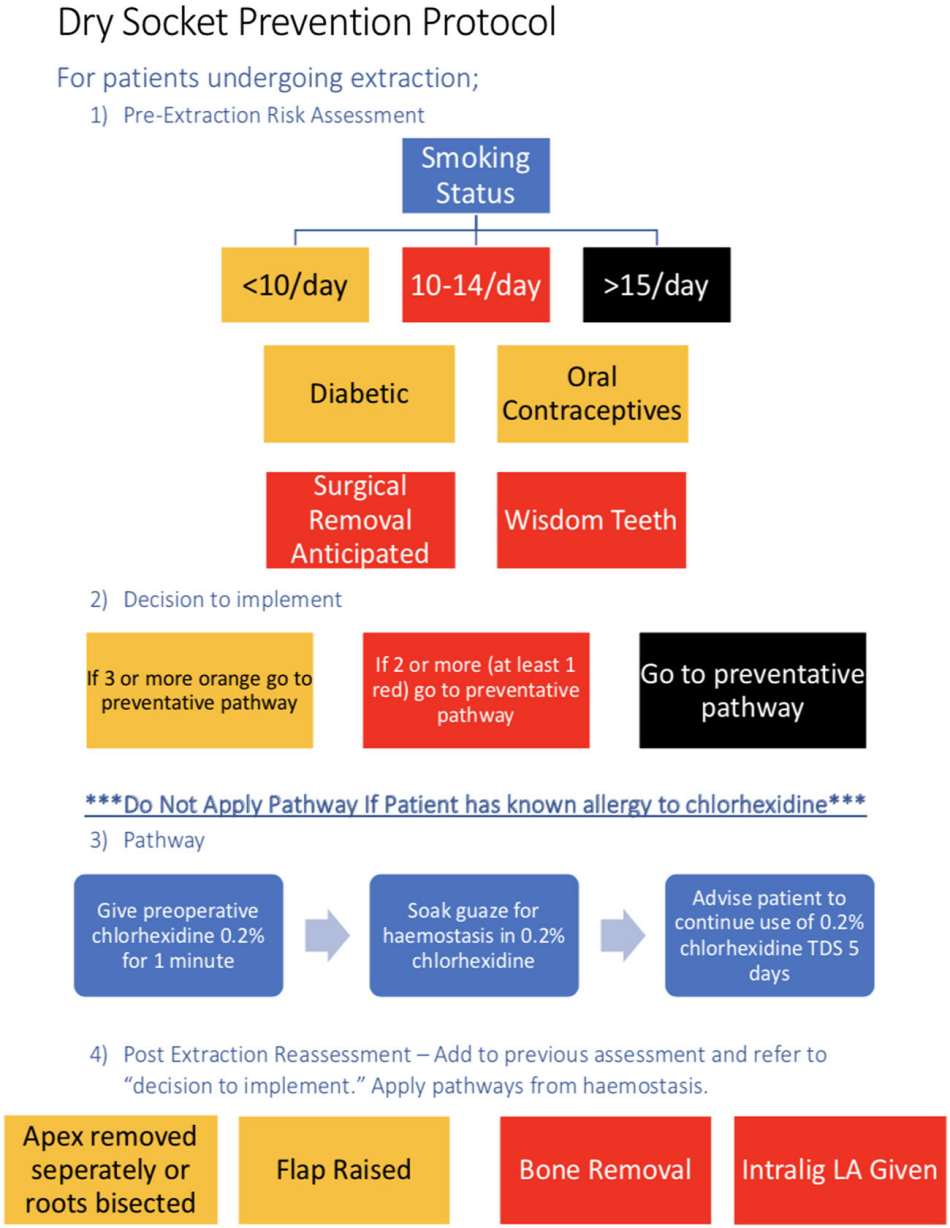
Dry socket prevention protocol

An intervention protocol was developed with the support and agreement of stakeholders, and implemented as part of this first PDSA cycle (see Fig. 3). This intervention was designed to be utilised alongside a routine extraction appointment with simple checks being required in the pre-, peri- and post-operative periods. If the patient was identified as high risk prior to extraction, the protocol recommended the use of 0.2% CHx mouthwash for 60 s prior to the commencement of extraction. Should the patient have already been identified as high risk, or should the extraction introduce risk factors (such as root fractures, or surgical removal of teeth), the protocol then recommended that the gauze used to achieve haemostasis be soaked in 0.2% chlorhexidine. It was also recommended that these patients be advised to continue with chlorhexidine mouthwash for the following 5 days. It was stressed to patients that this should bathe the area rather than actively rinsing or swilling as it was felt that this risked dislodging the clot and increasing the risk of AAO. It was also felt that compliance to homecare instructions was likely to be poor due to the necessity that patients actualise the advice and the potential need to actively purchase chlorhexidine mouthwash.

### Study of the intervention—measures and analysis

Clinical notes were the main focus for measuring the efficacy of the intervention. Clinicians were asked to record where the intervention had been utilised and reasons why it had been excluded were appropriate. The key outcome measure was the overall rate of AAO. Staff were also invited to give regular feedback on perceived strengths and weaknesses, and utilisation of the protocol was reviewed in staff meetings.

It was recognised that there was significant weekly variation in the pre-intervention data, and so 10 weeks were included to attempt to minimise this. A statistical process chart was developed to identify whether new data points were outside the control limits previously set, and hence infer the likelihood that a true shift in the trend was being seen.

### Ethical considerations

As this was a service improvement project, it did not require formal ethical approval. This was agreed by the Dental Teaching Unit and the Quality Improvement Skills Training Section of Health Education and Improvement Wales. Anonymous data were collected to ensure data protection and confidentiality.

Further consideration was given to the risk of acute anaphylactic reaction to perioperative CHx mouthwash. The rate of allergy to CHx within the UK is unknown although ranges between 0.5 and 2%, with higher rates reported in individuals with known anaphylactic reactions or eczema with 5% of these groups having positive patch response.^12^ As such it was decided that any individuals with a known allergy to CHx would be exempted from the protocol. It was also recommended that additional care would be taken for patients with other known anaphylactic reactions or eczema.

## Results

**Fig. 4:**
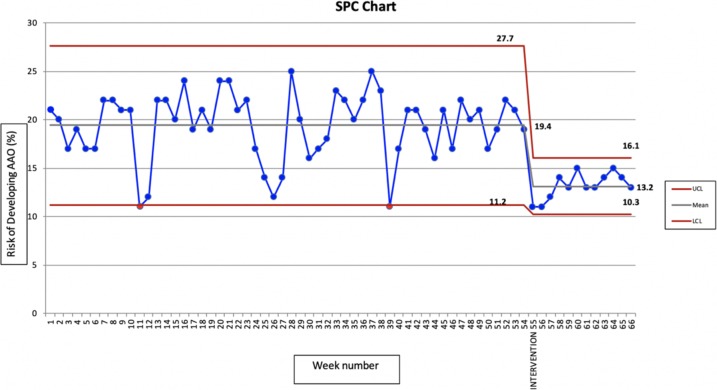
Statistical process chart for AAO

No alterations were made to the protocol following implementation, and the weekly rates of dry socket before and after intervention are displayed in Fig. 4.

It became apparent in reviewing clinical notes that there was significant variation in how and when it was recorded that the protocol was utilised. Whilst attempts were made to reinforce the importance of recording, this information to clinicians and at staff meetings these proved to be ineffective. As such, the number of the times the protocol was utilised or excluded had to be abandoned as an outcome measure. Whilst the clinicians were supportive and anecdotally engaged with the protocol, this highlights the importance of full stakeholder engagement throughout the quality improvement project and the difficulties of data collection in the dynamic-active clinical environment.

There was also compounding factors to the results, as coincident quality improvement projects were undertaken by other members of staff. Whilst the majority of these were not felt to be relevant to AAO, a coincident smoking-cessation project potentially increased the efficacy of utilising CHx. In review of the results, however, it was felt that the impact of this is likely reduced as it would only impact on regular patients who made up the minority of extractions conducted.

In total, 250 patients attended for extraction in the 10 weeks following implementation of the protocol. The statistical process chart showed a significant reduction in the mean and upper control limit, with a minor reduction in the lower control limit. Whilst an overall reduction of 6.2% and a relative reduction of 32% were considered clinically significant, the rate of AAO within the unit was still above the reported rate in the literature.

On review of the clinical notes for extraction appointments, 82 (32.8%) were deemed to have met the protocol guidance for the use of CHx preventative measures, however, it was clearly recorded in the notes that CHx had been given in 12 (14.6%) of these cases. CHx was not recorded as used in any other extraction appointments. In all, 33 (13.2%) patients were treated for AAO within 2 weeks of their extraction appointment.

## Discussion

Whilst record keeping was identified as an issue, there were no reported adverse events, no reported or anecdotal optional patient opt-outs of cases where patients did not given consent for the intervention, and anecdotally a high level of utilisation of the protocol. Whilst the intervention was initially designed to require minimal changes from routine practice, the limitations regarding data collection were significant. Consequently, a new data collection form was designed and distributed to clinicians to complete following extraction appointments with the intention that this would increase compliance and recording of the use of the protocol. This was initially met with resistance from clinicians due to increasing workloads, however, following feedback of the results there was general recognition for the value of this.

Whilst there are potentially compounding factors including increased clinicians’ awareness of risk factors leading to changes to case selection and concurrent quality improvement initiatives, it was felt given the primarily urgent nature of the extractions that the reduction seen was due directly or indirectly to the intervention. Furthermore, the reduction seen was in keeping with the reported effect of CHx in the literature, further supporting the efficacy in this case of the intervention. A cost analysis was undertaken, based on the absolute risk difference of 6.2%, the number needed to treat to prevent 1 case of AAO was 17. Within local ordering, 0.2% CHx costs £2.09/300 ml, resulting in a cost of £0.21 per patient treated with this protocol. Comparatively, within the DTU an emergency appointment usually equates to 15 min of clinical time equating to an estimated cost of £13.78, including peripheral staff and equipment costs. This equates to a net saving of ~£10.21 for each case prevented. It also increases availability of clinical time and increases opportunities for access to the service.

### Strengths and limitations

Whilst it was felt overall that the project resulted in a positive outcome, several key limitations are recognised. These include the incomplete data collection limiting the conclusion of how often the protocol was implemented, and the risk of concurrent projects compounding the result. Gaining insight into the patient experience of the intervention would have been beneficial regarding how acceptable they found the use of CHx. That being said, under the framework of Model for Improvement, the project recognised the dynamic and changing clinical environment and allowed for the introduction of changes recognising the limitations of clinical practice. This resulted in sustainable change to daily practice and saw a significant reduction in the rate of AAO.

It is recognised that the results of this project are highly specific to the local clinical environment and should be generalised with care and consideration of the high rate of local risk factors. For those considering replication of this intervention, understanding local context will be key to its safe and successful implementation.

## Conclusion

A range of quality improvement tools were utilised throughout this project to aid in identifying relevant variables. The identified intervention was advantageous in its simplicity to implement and availability of resources. The support of local stakeholders and clinical leaders was considered vital to the overall success of the project, however, in the current climate of “evidence-based practice,” the Model for Improvement allows clinicians to recognise the highly variable nature of different clinics, patient base and managerial styles, on the provision of care.^5^ It can support clinicians to select the most appropriate policies and, through a structured framework, assess whether these changes match with clinical need and result in improved outcomes.
